# Evaluation of the contribution of trio-exome sequencing in selected prenatal indications

**DOI:** 10.3389/fgene.2026.1761449

**Published:** 2026-05-11

**Authors:** Manon Chretien, Julien Osouf, Carine Abel, Alexandra Afenjar, Tania Attie-Bitach, Elise Brischoux-Boucher, Lydie Burglen, Nadège Calmels, Nicolas Chassaing, Thomas Courtin, Julian Delanne, Martine Doco-Fenzy, Christèle Dubourg, Benjamin Durand, Salima El Chehadeh, Laurence Faivre, Aurore Garde, Emmanuelle Ginglinger, Virginie Haushalter, Damien Haye, Solveig Heide, Laurence Heidet, Delphine Heron, Clémence Jacquin, Laetitia Lambert, Jean-Baptiste Lamouche, Vincent Laugel, Antony Le Bechec, Daphné Lehalle, Laurence Michel-Calemard, Edgar Montoya Ramirez, Jean Muller, Sylvie Odent, Olivier Patat, Juliette Piard, Céline Poirsier, Audrey Putoux, Chloé Quelin, Caroline Racine, Nicolas Sananes, Audrey Schalk, Sophie Scheidecker, Christel Thauvin-Robinet, Stéphanie Valence, Anne-Sophie Weingertner, Justine Wourms, Hélène Dollfus, Bénédicte Gerard, Caroline Schluth-Bolard, Elise Schaefer

**Affiliations:** 1 Service de Génétique Médicale, IGMA, CRBS, CHU de Strasbourg, Strasbourg, France; 2 Laboratoire de Diagnostic Génétique, Nouvel Hôpital Civil, CHU de Strasbourg, Strasbourg, France; 3 Service de Génétique, Groupement Hospitalier Est, Hospices Civils de Lyon, Bron, France; 4 Unité de Génétique Clinique et CRMR Malformations et Maladies Congénitales du Cervelet, Hôpital d’Enfants Armand-Trousseau, APHP, Paris, France; 5 Service de Médecine Génomique des Maladies Rares, Hôpital Necker-Enfants Malades, APHP, Paris, France; 6 Centre de génétique humaine, CHU Besançon, Université Franche-Comté, Besançon, France; 7 Laboratoire de Neurogénétique Pédiatrique et CRMR Malformations et Maladies Congénitales du Cervelet, Hôpital d’Enfants Armand-Trousseau, APHP Sorbonne Université, Paris, France; 8 Service de Génétique Médicale, Hôpital de Purpan, CHU de Toulouse, Toulouse, France; 9 Service de Génétique Clinique et Médicale, Hôpital de la Pitié Salpêtrière, APHP, Paris, France; 10 Université Bourgogne Europe, CHU Dijon Bourgogne, Inserm, CTM UMR1231, équipe GAD, FHU TRANSLAD, Centre de génétique, Centre de référence Anomalies du Développement et Syndromes Malformatifs, Centre de référence Déficiences Intellectuelles de Causes Rares, Dijon, France; 11 Service de Génétique, Hôpital Maison Blanche, CHU de Reims, Reims, France; 12 Service de Génétique, Hôtel Dieu, CHU de Nantes, Nantes, France; 13 Service de Génétique Moléculaire et Génomique Médicale, Hôpital Pontchaillou, CHU de Rennes, Rennes, France; 14 Service de Génétique Médicale, Hôpital Emile Muller, GHRMSA, Mulhouse, France; 15 Service de Néphrologie Pédiatrique, Centre de Référence des Maladies Rénales Héréditaires de l’Enfant et de l’Adulte (MARHEA), Hôpital Necker-Enfants Malades, APHP-centre, Université Paris Cité, Paris, France; 16 Laboratoire des Maladies Rénales Héréditaires, Inserm UMR 1163, Institut Imagine, Paris, France; 17 Unité de Consultation de Génétique, Hôpital d’enfants, CHU Reims, Reims, France; 18 Service de Génétique Clinique, Pôle Enfants Néonatalogie, CHRU de Nancy, Nancy, France; 19 INSERM-U1256 NGERE, Université de Lorraine, Nancy, France; 20 Unité de Bio-informatique Médicale Appliquée au Diagnostic, UF7363, Hôpitaux Universitaires de Strasbourg, Strasbourg, France; 21 Service de Pédiatrie 1, Hôpital de Hautepierre, CHU de Strasbourg, Strasbourg, France; 22 Service de Biochimie et Biologie Moléculaire, Centre de Biologie et Pathologie Est, Hospices Civils de Lyon, Bron, France; 23 Service d’Obstétrique, Hôpital du Hasenrein, GHRMSA, Mulhouse, France; 24 Laboratoire de Génétique Médicale, INSERM UMRS_1112, Université de Strasbourg, IGMA, CRBS, Strasbourg, France; 25 Service de Génétique Clinique, Hôpital Sud, CHU de Rennes, Rennes, France; 26 UMR 1231 GAD, Inserm, Université de Bourgogne, Dijon, France; 27 INSERM UMR1231, Equipe GAD, Université de Bourgogne Europe, Dijon, France; 28 Service de Gynécologie obstétrique, CMCO, CHU de Strasbourg, Strasbourg, France; 29 Département de Médecine Fœtale, Centre Pluridisciplinaire de Diagnostic PréNatal, CMCO, CHU de Strasbourg, Strasbourg, France; 30 Université Bourgogne Europe, CHU Dijon Bourgogne, Laboratoire de Génomique Médicale, Centre Neomics, FHU TRANSLAD, Centre de recherche Translationnelle en Médecine moléculaire - Inserm UMR1231, équipe GAD, Dijon, France; 31 Service de Neuropédiatrie, Hôpital d’Enfants Armand-Trousseau, APHP, Paris, France

**Keywords:** diagnostic yields, dual diagnosis, exome, incomplete phenotype, malformations, prenatal, somatic mosaic variants

## Abstract

**Objective:**

This study is an example of the contribution of exome sequencing (ES) in selected prenatal indications, while illustrating the complexity of interpreting prenatal genetic testing. Therefore, one of the aims of this study was to better describe antenatal phenotypes.

**Methods:**

This was a multicenter, comparative, prospective study assessing high-throughput sequencing performance (targeted gene panel *versus* exome sequencing) in selected prenatal indications.

**Results:**

Trio-ES was performed on 86 fetuses, allowed making a definite etiological diagnosis in 28% of cases (24/86). One-third of diagnoses were obtained only after exome analysis (8/24, 33%). The diagnostic yield varied according to the indication and was the highest for vermian hypoplasia (5/12, 42%) and polymalformative syndromes (30%, 8/27) indications. The variants identified were mainly missense variants in ciliopathy (18%, 6/33) and cytoskeleton (15%, 5/33) genes. Among the etiological diagnoses, one fetus carried a postzygotic mosaic variant in *RHOA*.

**Conclusion:**

In selected indications, the diagnostic yield of ES was close to 30% and varied according to the indications, showing the importance of clearly define the malformations justifying this approach. An etiological diagnosis was made in 23 families, within a timeframe compatible with pregnancy, thus improving the management of the pregnancy and/or the unborn child.

## Introduction

The risk of congenital malformation(s) during pregnancy is estimated at 2%–3% (Fu et al., 2018). It is the leading cause of morbidity and mortality during the perinatal period (between the 22nd week of amenorrhea (WA) and the 7^th^ day of life), and may be of toxic, infectious or genetic origin (EUROCAT, 2004). According to the EUROCAT register (https://eu-rd-platform.jrc.ec.europa.eu/eurocat_en), 2.8% of births were concerned between 2014 and 2024, the most common malformations being cardiac anomalies, particularly of the interventricular septum, club feet and polydactyly, congenital hydronephrosis and neural tube defects (Boyd et al., 2011; Tucker et al., 2018). An appropriate etiological workup should therefore be performed to characterize these anomalies. As part of the standardized diagnostic approach, when isolated or multiple anomalies are present and a strong diagnostic hypothesis is lacking, a chromosomal microarray analysis (CMA) remains the reference test, allowing identifying genomic imbalances with an overall diagnostic yield of 3%–5%, and up to 8% when multiple anomalies are present (Mastromoro et al., 2022). Given the great genetic heterogeneity and incomplete or scarce clinical information available during pregnancy, high-throughput sequencing (HTS) has proved to be a valuable tool, especially exome sequencing (ES). An analysis of five recent (2018-2019) antenatal cohorts that were globally homogeneous in terms of size and inclusion criteria (with 1,287 fetuses studied by ES) has shown a mean overall diagnostic yield of 19%. Fetuses with muscular or musculoskeletal anomalies had the highest diagnostic yield (32%), followed by urogenital anomalies (27.5%) and renal agenesis (25%). Fetuses with hydrops or renal, spinal or gastrointestinal anomalies had the lowest diagnostic yield (9%, 5.3%, 5% and 0,6% respectively) (Fu et al., 2018; Lord et al., 2019; Petrovski et al., 2019; Normand et al., 2018; Boissel et al., 2018) (Supplementary Data Sheet 1). Antenatal ES presents several difficulties, particularly with regard to incomplete phenotypes and/or phenotypes that evolve with the stage of pregnancy and are rarely described in the literature. Identifying variants of uncertain significance (VUS) or discovering incidental findings (IF) often requires discussion between the various stakeholders involved in prenatal diagnosis, within the short timeframe of pregnancy (Tran Mau-Them et al., 2023; Lefebvre et al., 2021). The current study is an example of the contribution of ES in selected prenatal indications, particularly those for which CMA yield was the lowest, with specific and unambiguous signs on morphological workup. The aim of this study was to describe and categorize phenotypes, and to enrich antenatal data on known and rarer syndromes.

## Materials and methods

### Study design and participants

This was a prospective, comparative, crossover study conducted in the genetic units and Multidisciplinary Centers for PreNatal Diagnosis of ten French university hospitals (Besançon, Dijon, La Pitié-Salpétrière, Lyon, Mulhouse, Nancy, Necker, Reims, Rennes, Strasbourg and Toulouse).

This study was approved on 5 June 2020 by the local Institutional Review Board (Comité de Protection des Personnes EST IV) under number N_DC-20142222. The research complied with the tenets of the Declaration of Helsinki. The consent of both members of the couple was obtained at the time of their inclusion, specifying that the results could be published in a scientific journal.

Inclusion criteria were:

- Ongoing pregnancy between 12 and 34 WA,
- Fetal DNA and DNA from both parents available for ES (1 µg minimum),
- Evidence of fetal malformation on ultrasound relevant to the selected indications. The following ultrasound signs, for which we had expertise and strongly suggestive of a monogenic disorder, were eligible for this study:
- Vermian hypoplasia, excluding Dandy Walker malformation, either isolated or associated with other cerebral disorders (microcephaly, lissencephaly, brainstem hypoplasia, etc.);
- Abnormal gyration associated with microcephaly;
- Isolated or syndromic (excluding trisomy 13) midline defect, including alobar, semi-lobar or lobar holoprosencephaly;
- Agenesis of corpus callosum (ACC);
- Severe unilateral or bilateral microphthalmia, vitreous hyperplasia;
- Isolated or syndromic large hyperechogenic kidneys, without associated urinary tract abnormalities;
- Non-regressive first-trimester hygroma colli or generalized hydrops;
- Polymalformative syndrome, including at least one major malformation and another ultrasound sign suggestive of an underlying genetic syndrome (major malformation, minor malformation, hydramnios, etc.).

For brain anomalies and polymalformative syndromes, fetal images were reviewed by an expert before confirming inclusion (Supplementary Data Sheet 1).

- Both members of the couple aged 18 years or over at the time of signing the consent form, and affiliated to a social health insurance scheme,
- Possibility of providing the couple with clear information and obtaining their written consent (no member of the couple under legal protection, guardianship or curatorship),
- Absence of aneuploidy on chromosomes 13, 18, 21 and X (systematic screening by PCR or FISH).

All included cases underwent a CMA, performed prior to or in parallel with HTS, depending on the participating centers.

### DNA extraction

Parental DNA was extracted from a blood sample according to standard procedures. Fetal DNA was extracted from chorionic villi or amniotic fluid, either native or in culture, using the QIAamp DNA mini kit (Qiagen, Hilden, Germany). The extracted DNA was then quantified using a Nanodrop spectrophotometer. The absence of maternal contamination was confirmed using the QSTRplusv2 kit (Elucigen, Manchester, United Kingdom) or Powerplex16 kit (Promega, Madison, WI, United States), according to the manufacturer’s instructions.

### Exome sequencing

Between 50 and 200 ng of DNA were used to prepare the exome library using Twist kit v7 design (Twist, San Francisco, CA, United States) or SureSelect XT HS2 Human All Exon V8+NCV (Agilent, Santa Clara, CA, United States). Samples were sequenced on a NextSeq550 sequencer (Illumina, San Diego, CA, United States) using the Illumina High Output Reagent Kit v2.0 or v2.5 (2*150 bp). This kit generates 800 million paired end reads and 120 Gb of data. Trio-ES was performed in the fetus and both parents. For 50 fetuses, maternal and paternal DNAs were pooled, allowing loading 5 index cases instead of 3 onto the same flow cell. Identity was verified by SNP (Single Nucleotide Polymorphism) analysis, with amplification by Taqman assay using a LightCycler 480 (Roche, Basel, Switzerland) in parallel with ES (Supplementary Data Sheet 1).

### Bioinformatics analysis

After sequencing, reads (800 million reads on average) were analyzed using our in-house pipeline, STARK (v18.3) (Stellar Tools from raw sequencing data analysis to variant RanKing). Reads were aligned against the reference human genome (GRCh37/hg19) using BWA-MEM(12) (v0.7.17). SNV (Single Nucleotide Variation) and indel calling was performed using both the HaplotypeCaller and UnifiedGenotyper programs from the Genome Analysis Toolkit (GATK) (Van der Auwera et al., 2013) v.3.8. Annotation and ranking of SNVs and indels were performed using VaRank (v1.4.3) (Geoffroy et al., 2015) and Alamut Batch (v1.11) (Sophia Genetics). Copy Number Variants (CNVs) were called using CANOES (Backenroth et al., 2014) (v1.0) and annotated and ranked using AnnotSV (Geoffroy et al., 2023) (v3.0). Mitochondrial DNA, balanced rearrangements, mobile elements, triplet amplifications were not explored. The following databases were used to interpret variant significance, including patients’/pathogenic variant databases (HGMDpro (Stenson et al., 2003) /ClinVar (Landrum et al., 2018)), population database (gnomAD (Gudmundsson et al., 2022) v2.1.1, 1,000 genomes, local internal database including 300 index cases and relatives) and OMIM (Amberger and Hamosh, 2017). Prediction tools for missense variants included PolyPhen-2, SIFT (Kumar et al., 2009), MutationTaster (Schwarz et al., 2010) and physicochemical changes while the effects on splicing was assessed for synonymous, missense and intronic variants using MaxEntScan, NNSplice and Splice Site Finder (Shapiro and Senapathy, 1987; Yeo and Burge, 2004; Reese et al., 1997) (Supplementary Data Sheet 1).

The study was conducted in two steps. Step 1 was limited to a targeted gene panel relevant to the indication-specific inclusion criteria for each patient (*in silico* panel). Gene lists were validated by the expert laboratory and the healthcare networks concerned (Supplementary Table S1). Step 2 corresponded to the extension of the analysis to the whole exome.

Variants were classified according to the American College of Medical Genetics (ACMG) criteria (Rich et al., 2015). Any likely pathogenic (class 4) or pathogenic (class 5) variant that was related to the phenotype of the fetus was examined by a second biologist before being returned to the expert clinician.

All VUSs and IF were discussed between the laboratory experts and the clinician(s).

## Results

### Characteristics of the participants

**FIGURE 1:**
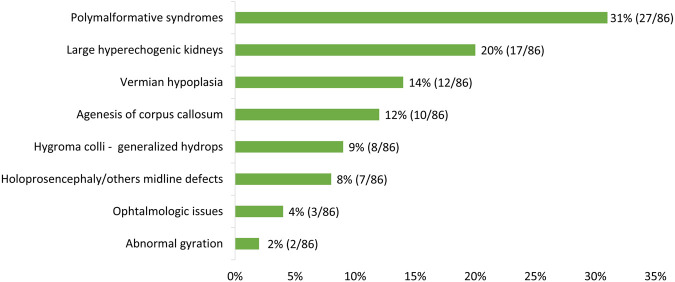
Fetuses’ distribution according to the clinical indication (no overlap: only one indication per fetus).

Eighty-six fetuses were included and analyzed in this study. The mean term of pregnancy at the time of inclusion was 22 WA (range: 12–33 WA), with, as expected, earlier discovery of cases with hygroma colli/generalized hydrops and holoprosencephaly/other midline defects (mean: 17 WA and 18 WA, respectively) and later discovery of cases with ophthalmologic issues and abnormal gyration (27 WA and 29 WA, respectively) (Supplementary Table S2). The most common indications were polymalformative syndromes (27/86, 31%) and large hyperechogenic kidneys (17/86, 20%) (Figure 1).

### Diagnostic yields

#### Overall yields

Trio-ES was performed in 86 fetuses and allowed identifying ACMG class 4 or 5 variants, leading to an etiological diagnosis in 28% of cases (24/86). Of these 24 cases, a positive diagnosis was made by primary targeted gene panel analysis in 16 cases (i.e. 2/3 of the cases with a positive diagnosis or 19% of the 86 cases analyzed), while 8 cases were diagnosed (*PIK3R2*, *ASPM*, *PLK4*, *TRIP4*, *RHOA*, *CREBBP*, *DCC* and *CHRNG* genes) by ES (i.e. 1/3 of cases that underwent additional diagnostic analysis or 9% of the 86 cases analyzed) (Table 1).

**TABLE 1:** Diagnostic yields according to the type of indication.

Indication	Number of fetuses	Definitive diagnosis (ACMG class 4/5)	Definitive diagnostic yield (%)	Genes	Potential diagnosis (ACMG class 3)	Potential diagnostic yield (%)	Genes
Polymalformative syndromes	27	8	30	*CHD7* *QRICH1* *DHCR7* *CHRNG* *KMT2D* *DDX3X*	3	11	*LZTR1* *ALG3*
Large hyperechogenic kidneys	17	4	23	*PKD1* *CEP290* *BBS1* *HNF1B*	3	18	*PKD1* *PKD2*
Vermian hypoplasia	12	5	42	*PIK3R2* *DYNC1H1* *CREBBP, KIAA0586* *ASPM*	0	0	​
ACC	10	2	20	*ARID1B* *DCC*	1	10	*DCC*
Hygroma colli/generalized hydrops	8	2	25	*TRIP4* *LZTR1*	2	25	*MAST1* *THSD1*
Holoprosencephaly/other midline defects	7	1	14	*ZIC2*	1	14	*TUBB*
Ophthalmologic issues	3	1	33	*RHOA*	0	0	​
Abnormal gyration	2	1	50	*PLK4*	0	0	​
Total	86	24	28	​	10	**12**	​

ACMG class 3 variants (VUSs) were found in 11.6% of cases (10/86). Of these cases, 30% were identified by targeted gene panel (i.e. 3/10 or 3.5% of the 86 cases analyzed, in the *PKD1* and *PKD2* genes) but 70% of VUSs (in the *TUBB*, *MAST1*, *LZTR1*, *ALG3*, *THSD1* and *DCC* genes) were identified by ES (i.e. 7/10 or 8% of the 86 cases analyzed) (Table 1).

### Diagnostic yield according to the indication

The highest diagnostic yields (class 4/5) were obtained for fetuses with the following indications: vermian hypoplasia (42%, 5/12), polymalformative syndromes (30%, 8/27), hygroma colli/generalized hydrops (25%, 2/8) and large hyperechogenic kidneys (24%, 4/17) (Table 1).

VUSs were mainly found in fetuses with the following indications: large hyperechogenic kidneys (17.5%, 3/17), hygroma colli/generalized hydrops (25%, 2/8) and polymalformative syndromes (11%, 3/27) (Table 1).

### Type of variants, inheritance and biological functions

#### Variant type (ACMG class 3, 4 and 5)

The variants identified were mainly missense variants (20/39, 51%). Additionally, nine non-sense variants (23%), seven frameshift variants (18%), one splice variant (3%) and 2 CNV (5%) were identified. Of these, only the CNV in the *HNF1B* gene could be identified by CMA (006-003, renal cysts and diabetes syndrome, #137920) (Supplementary Table S2).

#### Inheritance (ACMG class 3, 4 and 5)

**FIGURE 2:**
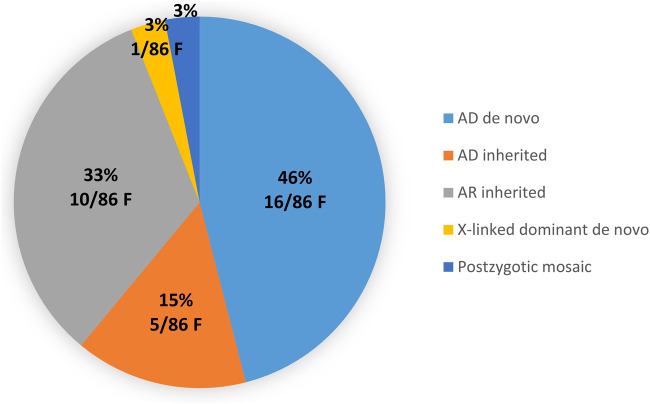
Distribution of the variants according to the inheritance mode in all families (ACMG class 3, 4 and 5). F: Fetuses.

Most variants were transmitted in an autosomal dominant (AD) manner (61%, 20/33), with 46% (15/33) of *de novo* variants and 15% (5/33) of variants transmitted by a paucisymptomatic *(TUBB)* or asymptomatic parent (*DCC*, *PKD1*). Autosomal recessive (AR) variants were found in 33% of cases (11/33). Only one X-linked disease (dominant, *de novo* in *DDX3X*) was diagnosed. Finally, a postzygotic mosaic variant (1/33, 13%) was found in the *RHOA* gene (Figure 2; Table 2; Table 3; Supplementary Table S2).

**TABLE 2:** Definitive diagnosis: ACMG class 4 and 5 variants.

​	Patient ID	Prenatal phenotype	ACMG class	Gene	Chr	NM_ Transcript reference	c. Nucleotide variation p. Protein variation	Transmission	Inheritance	Status	Phenotype MIM number/Biological function involved (UniProtKB)
Polymalformative syndromes	009–004	Abnormal heart valve morphology, hydramnios Abnormal external genitalia	5	*CHD7*	8	NM_017780.4	c.469C>T p. (Arg157*)	AD	*de novo*	Heterozygote	CHARGE syndrome #214800/DNA metabolism
	001–011	Hygroma colli, cleft lip and palate, tetralogy of fallot	4	*CHD7*	8	NM_017780.4	c.2097–2A>G p.?	AD	*de novo*	Heterozygote	CHARGE syndrome #214800/DNA metabolism
	001–015	Hygroma colli, IUGR, microphthalmia, cleft lip and palate Tetralogy of fallot	5	*CHD7*	8	NM_017780.4	c.6157C>T p. (Arg 2053*)	AD	*de novo*	Heterozygote	CHARGE syndrome #214800/DNA metabolism
	009–007	Hygroma colli, abnormal upper and lower extremities Decreased fetal movements	5	*CHRNG*	2	NM_005199.5	c.459dup p. (Val154Serfs *24)	AR	Mat + pat	Homozygote	ESCOBAR syndrome #265000/Canalopathy
	001–032	Hygroma colli, severe IUGR	5	*DDX3X*	X	NM_001193416.3	c.1703C>T p. (Pro568Leu)	X-linked	*de novo*	Hemizygote	Intellectual developmental disorder, X-linked syndromic, snijders blok type #300958/DNA metabolism
	008–011	Hygroma colli, IUGR, ASD, short CC, Hypoplastic hyperechogenic kidneys	5	*DHCR7*	11	NM_001360.2	c.440G>A p. (Gly147Asp)	AR	Mat + pat	Homozygote	Smith-lemli-opitz syndrome #270400/Other signaling pathways
	009–008	Fetal hydrothorax, microcephaly, short femur	4	*KMT2D*	12	NM_003482.4	c.16391C>T p. (Thr5464Met)	AD	*de novo*	Heterozygote	Kabuki syndrome 1 #147920/Chromatinopathy
	001–013	Hygroma colli, hydrocephalus	4	*QRICH1*	3	NM_017730.3	c.1647C>G p. (Tyr549*)	AD	*de novo*	Heterozygote	Ververi-brady syndrome #617982/DNA metabolism
Large hyperechogenic kidneys	003–003	Bilateral nephromegaly, hexadactyly	5	*BBS1*	11	NM_024649.4	c.1169T>G p. (Met390Arg)	AR	Mat + pat	Homozygote	Bardet-biedl syndrome 1 #209900/Ciliopathy
	008–003	Large hyperechogenic kidneys, anamnios, enlarged ventricles	5	*CEP290*	12	NM_025114.3	c.1078C>T p. (Arg360*)	AR	Mat	Compound heterozygote	Numerous ciliopathies such as: Joubert syndrome 5 #610188; meckel syndrome 4 #611134; bardet-biedl syndrome 14 #615991/Ciliopathy
			5	*CEP290*	12	NM_025114.3	c.5493del p. (Ala1832Profs*19)	AR	Pat	Compound heterozygote	
​	006–003	Large hyperechogenic kidneys	5	*HNF1B* 17q12del	17	NM_000458.4	c. [-175-?_*942+?] p. [0]	AD	NA	Heterozygote	Renal cysts and diabetes syndrome #137920/Ciliopathy
	001–004	Hygroma colli, large polycystic kidneys, hydramnios	5	*PKD1*	16	NM_001009944.2	c.2534T>C p. (Leu845Ser)	AD	*de novo*	Heterozygote	Polycystic kidney disease 1 #173900/Ciliopathy
Vermian hypoplasia	003–002	Microcephaly, simplified gyral pattern Brainstem hypoplasia	5	*ASPM*	1	NM_018136.4	c.3130dup p. (Thr1044Asnfs*19)	AR	Pat	Compound heterozygote	Microcephaly 5, primary, autosomal recessive #608716/Cytoskeleton
			5	*ASPM*	1	NM_018136.4	c.6919C>T p. (Gln2307*)	AR	Mat	Compound heterozygote	
	002–003	Vermian hypoplasia	5	*CREBBP*	16	NM_004380.3	c.3832G>A p. (Glu1278Lys)	AD	*de novo*	Heterozygote	Rubinstein-taybi syndrome 1 #180849/Chromatinopathy
	008–006	Hydrops, cerebellar hypoplasia, thick CC	5	*DYNC1H1*	14	NM_001376.5	c.1738G>A p. (Glu580Lys)	AD	*de novo*	Heterozygote	Cortical dysplasia, complex, with other brain malformations 13 #614563/Cytoskeleton
	001–021	Enlarged cisterna magna, vermian hypoplasia	5	*KIAA0586*	14	NM_001329943.3	c.392del p. (Arg131Lysfs*4)	AR	Mat	Compound heterozygote	Joubert syndrome 23 #616490/Ciliopathy
			5	*KIAA0586*	14	NM_001329943.3	c.586-2_1129del p.?	AR	Pat	Compound heterozygote	
	003–001	Vermian hypoplasia, unilateral enlarged ventricle	4	*PIK3R2*	19	NM_005027.3	c.1054T>C p. (Phe352Leu)	AD	*de novo*	Heterozygote	Megalencephaly-polymicrogyria-polydactyly-hydrocephalus syndrome 1 #603387/Other signaling pathways
ACC	003–005	ACC, enlarged cisterna magna Absence of left superior vena cava	5	*ARID1B*	6	NM_001374828.1	c.6353_6356del p. (Tyr2118*)	AD	*de novo*	Heterozygote	Coffin-siris syndrome 1 #135900/Chromatinopathy
			5	*DCC*	18	NM_005215.3	c.601C>T p. (Arg201*)	AD	Pat	Heterozygote	Mirror movements 1 and/or ACC #157600/Neuronal migration
Hygroma colli - generalized hydrops	001–012	Generalized hydrops	4	*LZTR1*	22	NM_006767.4	c.1942G>T p. (Gly648Cys)	AR	Mat + pat	Homozygote	Noonan syndrome 2 #605275/Rasopathy
	008–009	Subcutaneous cephalic edema	5	*TRIP4*	15	NM_016213.4	c.832C>T p. (Arg278*)	AR	Mat + pat	Homozygote	Spinal muscular atrophy with congenital bone fractures 1 #616866/Transcription factor
Holoprosencephaly /Other midline defects	001–008	Holoprosencephaly, enlarged ventricles	4	*ZIC2*	13	NM_007129.5	c.998_999delinsCT p. (Phe333Ser)	AD	*de novo*	Heterozygote	Holoprosencephaly 5 #609637/Transcription factor
Ophthalmologic issues	006–001	Enlarged ventricles, unilateral anophthalmia	5	*RHOA*	3	NM_001664.4	c.139G>A p. (Glu47Lys)	​	*de novo*	Somatic mosaïc 13%	Ectodermal dysplasia with facial dysmorphism and acral, ocular and brain anomalies, somatic mosaic #618727/Cytoskeleton
Abnormal gyration	008–007	IUGR, microcephaly, simplified gyral pattern Hypoplastic CC	5	*PLK4*	4	NM_014264.5	c.1299_1303del p. (Phe433Leufs*6)	AR	Mat	Compound heterozygote	Microcephaly and chorioretinopathy, autosomal recessive, 2#616171/Centriole
			4	*PLK4*	4	NM_014264.5	c.2806A>C p. (Thr936Pro)	AR	Pat	Compound heterozygote	

ACC, agenesis of corpus callosum; AD, autosomal dominant; AR, autosomal recessive; ASD, atrial septal defect; CC, corpus callosum; Chr, Chromosome; HPA, human protein atlas; IUGR, intra uterine growth retardation; mat, maternal; NA, not available; pat, paternal.

**TABLE 3:** Potential diagnosis: ACMG class 3 variants.

​	Patient ID	Prenatal phenotype	ACMG class	Gene	Chr	NM_ Transcript reference	c. Nucleotide variation p. Protein variation	Transmission	Inheritance	Status	Phenotype MIM number/Biological function involved (UniProtKB)
Polymalformative syndromes	001–014	Choroid plexus cyst, enlarged sylvian cistern Cerebellar hypoplasia Cardiopathy, retrognathism	3	*ALG3*	3	NM_005787.6	c.1057A>C p. (Ser353Arg)	AR	Mat	Compound heterozygote	Congenital disorder of glycosylation, type id #601110/Protein glycosylation
			3	*ALG3*	3	NM_005787.6	c.1237C>T p. (His413Tyr)	AR	Pat	Compound heterozygote	
			3	*LZTR1*	22	NM_006767.3	c.1778_1784del p. (Gln593Argfs*5)	AR	Mat	Heterozygote	Noonan syndrome 2 #605275/Rasopathy
	001–024	Hygroma colli, fetal hydrothorax	3	*LZTR1*	22	NM_006767.3	c.1234C>T p. (Arg412Cys)	AD	*de novo*	Heterozygote	Noonan syndrome 10 #616564/Rasopathy
Large hyperechogenic kidneys	001–004	Large hyperechogenic multicystic kidneys Hygroma colli	3	*PKD1*	16	NM_001009944.2	c.11602A>G p. (Thr3868Ala)	AD	Mat	Heterozygote	Polycystic kidney disease 1 #173900/Ciliopathy
	004–001	Hyperechogenic kidneys	3	*PKD1*	16	NM_001009944.2	c.11948T>A p. (Leu3983Gln)	AD	Pat	Heterozygote	Polycystic kidney disease 1 #173900/Ciliopathy
	001–005	Large hyperechogenic kidneys	3	*PKD2*	4	NM_000297.4	c.2725G>C p. (Val909Leu)	AD	*de novo*	Heterozygote	Polycystic kidney disease 2 #613095/Ciliopathy
ACC	001–017	ACC	3	*DCC*	18	NM_005215.3	c.2689-1G>A p.?	AD	Mat	Heterozygote	Mirror movements 1 and/or ACC #157600/Neuronal migration
Hygroma colli -generalized hydrops	008–009	Persistent cephalic subcutaneous edema	3	*MAST1*	19	NM_014975.2	c.4423dup p. (Arg1475Profs*76)	AD	*de novo*	Heterozygote	Mega-corpus-callosum syndrome with cerebellar hypoplasia And cortical malformations #618273/Cytoskeleton
	001–016	Isolated fetal ascites	3	*THSD1*	13	NM_018676.4	c.670C>T p. (Arg224*)	AD	*de novo*	Heterozygote	Lymphatic malformation 13 #620244/Cytosol
Holoprosencephaly/Other midline defects	004–003	Thin CC, distortion of interhemispheric fissure	3	*TUBB*	6	NM_001293212.1	c.421C>T p. (Arg141Trp)	AD	Mat	Heterozygote	Cortical dysplasia, complex, with other brain malformations #615771/Cytoskeleton

ACC, agenesis of corpus callosum; AD, autosomal dominant; AR, autosomal recessive; CC, corpus callosum; Chr, Chromosome; HPA, human protein atlas; mat, maternal; pat, paternal.

#### Biological functions involved

**FIGURE 3:**
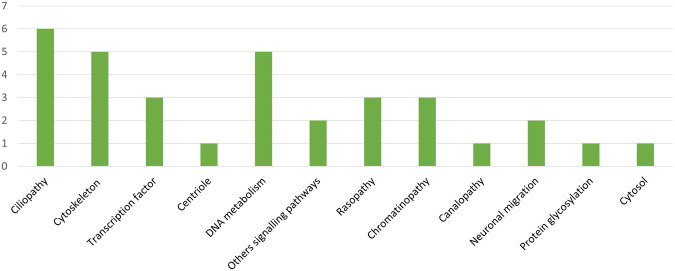
Biological functions affected by the mutated genes identified (ACMG class 3, 4 and 5 variants; Absolute value on ordinate). Classification of biological functions based on UniProtKB/uniprot.org/keywords using the Human Protein Atlas website/proteinatlas.org/.

Of the 33 cases with a distinct positive diagnosis or potential diagnosis (ACMG class 3, 4 or 5), the variants identified were mainly in ciliopathy genes (18%, 6/33), DNA metabolism genes (15%, 5/33) and cytoskeleton genes (15%, 5/33) (Table 2; Table 3; Figure 3).

In fetuses with large hyperechogenic kidneys, ACMG class 3, 4 or 5 variants were only found in ciliopathy genes (8/8, 100%). Only one case of ciliopathy was diagnosed in a fetus with cerebral anomalies (001-021, Joubert Syndrome 23 #616490). Moreover, almost half of the ACMG class 3, 4 or 5 variants identified in fetuses with polymalformative syndromes were in DNA metabolism genes (5/12, 42%). In fetuses with hygroma colli/generalized hydrops, only one case of rasopathy (001-012, 1/4, 25%) was identified, showing the lack of specificity of this clinical sign, despite the small number of cases included (Table 2; Table 3).

### Diagnosis

#### ACMG class 4 or 5 variants: pathogenic or likely pathogenic (P/LP) variants

ACMG class 4 or 5 variants (n = 28) were identified in 21 different genes, with three recurrences for the *CHD7* gene (CHARGE Syndrome #214800) (n = 3). Fetuses (009-004, 001-011 and 001-015) carrying a P/LP variant in the *CHD7* gene had a phenotype consistent with CHARGE syndrome, i.e., a combination of IUGR (IntraUterine Growth Retardation), tetralogy of Fallot, abnormal external genitalia, and cleft lip and palate, highlighting a strong genotype/phenotype correlation (Table 2).

The *PKD1*, *CEP290*, *BBS1* and *HNF1B* genes, associated with a ciliopathy phenotype, were mutated in fetuses with large hyperechogenic kidneys, highlighting again a strong genotype/phenotype correlation (Table 2).

#### ACMG class 3 variants: Variants of uncertain significance (VUSs)

VUSs (n = 11) were identified in 8 different genes with two recurrences for the *PKD1* (001-004, 004-001, Polycystic kidney disease 1 #173900) (n = 2) and *LZTR1* (001-024, 001-014, Noonan syndrome 10 #616564 and Noonan syndrome 2 #605275) (n = 2) genes. Note that the AR VUS in *LZTR1* (001-014, Noonan syndrome 2 #605275) was identified here based on the assumption of a second unknown variant (CDG syndrome not considered, no fetal autopsy). VUSs identified in the *PKD1* and *PKD2* genes could potentially explain all or part of the fetal phenotype (large hyperechogenic kidneys, 001-004, 004-001, 001-005) (Table 3).

### Dual diagnosis

After next-generation sequencing data analysis and interpretation, four fetuses carried two independent variants in two different genes that could be associated with the observed antenatal phenotype. Among them, three independent cases carried at least one VUS (008-009, 001-014 and 001-004) (Tables 2, 3).

Only one fetus (003-005) carried two ACMG class 5 variants in the *ARID1B* and *DCC* genes, suggesting a dual positive diagnosis. In this female fetus, ACC was detected at 22 WA (first pregnancy in an unrelated couple). Fetal CMA was unremarkable. Primary targeted gene panel analysis revealed a *de novo* ACMG class 5 variant in the *ARID1B* gene (Coffin-Siris syndrome, #135900): NM_001374828.1:c.6353_6356del; p.(Tyr2118*). Subsequent ES revealed an ACMG class 5 variant in the *DCC* gene (Mirror movements 1 and/or ACC, #157600), inherited from the father: NM_005215.3: c.601C>T; p.(Arg201*) (Table 2). The father was asymptomatic, with brain MRI findings within normal limits. The pregnancy was terminated at 29 WA due to a poor neurodevelopmental prognosis related to *ARID1B* variations. Macroscopic fetal examination revealed facial dysmorphia with slightly anteverted nares, microretrognatism and low-set ears (no consent to publish). Bilateral single transverse palmar crease was observed, with no clear brachydactyly. The feet showed distal brachydactyly with short nails. Post-mortem X-rays showed globally hypoplastic P3 phalanges of the toes and non-ossified P3 phalanges of the 5^th^ toes (X-rays not available). The couple had access to Non-Invasive Prenatal Diagnosis (NIPD), solely for the purpose of screening for the *ARID1B* variant for a subsequent pregnancy.

The two pathogenic variants identified in the *ARID1B* and *DCC* genes could explain ACC diagnosed in the fetus at 22 WA. Only the *ARID1B* variant could explain the morphological features (Martins-Costa et al., 2024; Vergano et al., 1993; Marsh et al., 2018).

### Illustration of the complexity of interpreting prenatal genetic testing

**FIGURE 4:**
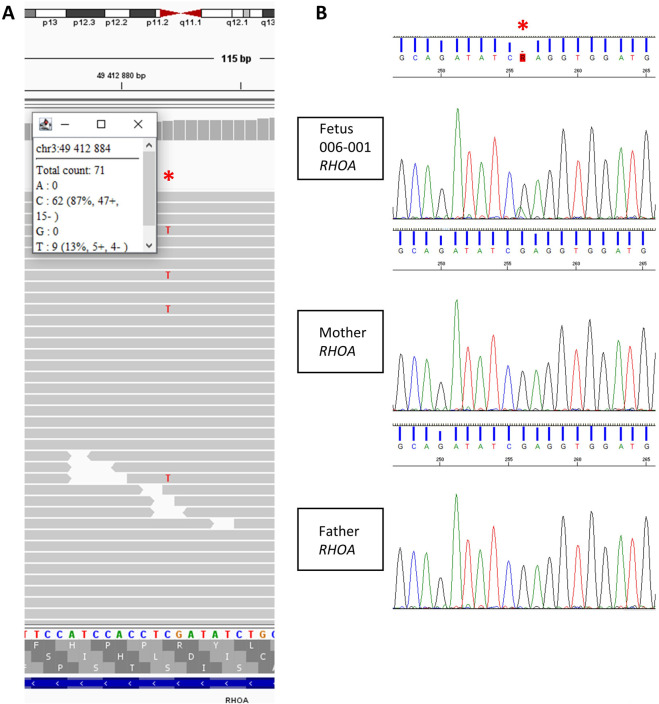
*RHOA* NM_001664.4 Chr3 (GRCh37):g.49412884C>T c.139G>A; p.(Glu47 Lys) *de novo* mosaicism (13%) in fetus 006-001. **(A)** Raw amniotic fluid exome sequencing data (*.bam), **(B)**. Sanger sequencing on amniotic fluid of fetus 006-001 and the blood of the parents.

The main difficulty in interpreting prenatal molecular data lies in the scarcity of data available in the literature on antenatal descriptions of phenotypes in rare syndromes. In addition, the antenatal phenotype of the cases to be analyzed is often incomplete due to ultrasound limitations, the stage of pregnancy, etc. Genotype/phenotype correlations are therefore difficult to establish. Below are two examples of positive diagnosis illustrating these difficulties:

Moderately enlarged ventricles and unilateral anophthalmia were detected in a fetus at 31 WA (006-001). Fetal CMA was unremarkable. ES revealed a pathogenic somatic mosaic variant (13%) in the *RHOA* gene: NM_001664.4: c.139G>A; p.(Glu47Lys), confirmed by Sanger sequencing on the same amniotic fluid sample (Table 2; Figures 4A,B). P/LP variants in *RHOA* are associated with a phenotype characterized by cerebral, ocular and acral anomalies with ectodermal dysplasia (#618727). In this context, the pregnancy was terminated at 33 WA. Fetopathological examination revealed unilateral right anophthalmia with meningeal congestion and bilateral ventricular dilation, as well as anomalies of the extremities with partial syndactyly of the 2^nd^ and 3^rd^ toes of the right foot and the 3^rd^ and 4^th^ toes of the left foot, supporting the molecular diagnosis.

A 4.9-mm hygroma colli was detected in a female fetus at 13 WA (001-032). Fetal CMA was unremarkable. The hygroma colli regressed but the fetus subsequently presented with severe IUGR with normal Doppler findings, justifying further genetic testing (in the “polymalformative syndrome” indication). A *de novo* ACMG class 5 hemizygote variant was identified in the *DDX3X* gene involved in “Intellectual developmental disorder, X-linked syndrome, Snijders Blok type” (#300958): NM_001193416.3: c.1703C>T; p.(Pro568Leu) (Table 2). The pregnancy was terminated at 32 WA + 6 days. No fetopathological examination was performed.

### Pregnancy outcome, the couple’s decision

The decision to continue the pregnancy varied according to the indication and the severity of the fetal phenotype. The presence of cerebral anomalies was more likely to lead to pregnancy termination, especially in case of vermian hypoplasia (83.3%, 10/12) and ACC (60%, 6/10). However, this decision did not appear to be related to the molecular diagnosis. In the vast majority of cases, pregnancy was terminated before HTS results were available (77%, 30/39), mainly in case of holoprosencephaly/other midline defects (100%, 3/3), large hyperechogenic kidneys (100%, 7/7), hygroma colli/generalized hydrops (100%, 2/2), polymalformative syndromes (82%, 9/11) and vermian hypoplasia (67%, 6/9) (Supplementary Table S2).

## Discussion

### Diagnostic yield close to 30%

Comparing different series of prenatal ES data remains challenging, due to multiple criteria influencing the results, including the cohort size and inclusion criteria, when the study was conducted, etc. Furthermore, the size of our cohort (both overall and by indication) does not allow for a meaningful statistical analysis. Compared to data from five previously published large prenatal cohorts (a total of 249 etiological diagnoses made in 1,287 fetuses) (Fu et al., 2018; Lord et al., 2019; Petrovski et al., 2019; Normand et al., 2018; Boissel et al., 2018), the results of this study are broadly in line with the literature in terms of diagnostic yield, type of disorders/biological functions involved, recurrent genes and associated phenotypes. Indeed, we found an overall diagnostic yield of about 30%, with mainly the identification of AD genes involved in the genesis or function of the primary cilium (ciliopathies) and in DNA metabolism. Ciliopathies were mainly diagnosed in individuals with large hyperechogenic kidneys (*PKD1, BBS1, HNF1B, CEP290*). In addition, the *CHD7* variants were recurrent (n = 3) in our cohort, in individuals with multiple malformations (tetralogy of Fallot, and cleft lip and palate). In this study, the diagnostic yield varied greatly according to the indications and therefore the phenotypic context (isolated or multiple anomalies, type of anomaly), and was higher in the presence of antenatal cerebral anomalies (vermian hypoplasia with other brain malformations, non-isolated ACC). A similar prospective multicenter French study from 2023 also reported an overall diagnostic yield of 34%. However, fetuses with isolated hygroma colli were not included, which, given the low diagnostic yield of this indication in exome sequencing, once again makes it difficult to compare the results with our cohort (Tran Mau-Them et al., 2023). It therefore seems essential to clearly define the antenatal malformations that justify the use of this approach.

## Contribution of exome sequencing

Compared to the use of targeted gene panels, and as previously demonstrated in the literature, this study highlighted the value of performing antenatal ES, especially when only non-specific isolated ultrasound findings were found, sometimes allowing identifying pathogenic variants in genes described more recently/involved in a particular molecular mechanism (e.g., somatic mosaic variants) (Tran Mau-Them et al., 2023; Lefebvre et al., 2021).

Also, the molecular diagnosis could influence the couple’s decision to continue the pregnancy. For example, variants in some genes may be associated with a better prognosis, such as variants in *DCC* associated with isolated ACC, with incomplete penetrance.

### Interpreting prenatal tests remains challenging

The emergency context of pregnancy adds to the difficulty of interpreting molecular data, especially when the analysis is carried out at an advanced stage of pregnancy.

The nature of the variant itself may or may not facilitate interpretation, notably in the case of previously undescribed missense variants, especially when the antenatal phenotype is partial. For example, in a fetus with hygroma colli and severe IUGR (001-032), a pathogenic missense variant was identified in late pregnancy in the *DDX3X* gene, which may be associated with a polymalformative phenotype (atrial or ventricular septal defect, cerebral anomalies of corpus callosum, white matter). The difficulty with this fetus lay in the absence of any clinical signs other than persistent IUGR in the third trimester, which is particularly nonspecific (Levy et al., 2023; Snijders et al., 2015; Johnson-Kerner et al., 1993; Lennox et al., 2020).

Incomplete penetrance and variable expressivity of a disorder in the context of a variant inherited from an asymptomatic or paucisymptomatic parent sometimes complicate the determination of a causal relationship. Also, in an antenatal context, two different phenotypic presentations may be associated with the same gene, reflecting variable tissue expressivity (e.g., renal or cerebral involvement for ciliopathy genes).

Finally, dual diagnosis raises questions, particularly when a hypomorphic allele inherited from an asymptomatic parent worsens the fetal phenotype (e.g., two heterozygote variants in *PKD1* with a severe antenatal phenotype, 001-004).

Since VUSs remain numerous even after ES, they need to be reinterpreted over time and therefore require postnatal monitoring or fetopathological examination (Tran Mau-Them et al., 2023). Attitudes towards the communication of these VUSs and their management in antenatal care differ according to the teams responsible for interpreting the analysis and according to the countries (Gabriel et al., 2022; Fu et al., 2022). In France, the consensus established by the Haute Autorité de Santé (HAS) does not allow such data to be disclosed before birth. If they are not reclassified, these VUSs will not allow making a prenatal diagnosis for a subsequent pregnancy.

ES also increases the risk of discovering IF (not reported in this study). In France, the revision of the bioethics law of 02 August 2021 has permitted the communication of IF to the patient (amendment of article 16-10 of the civil code and article 2131-1 of the public health code). In the antenatal context, opinions on the disclosure of IF to parents differ. No consensus has yet been reached.

## Conclusion

Between 2020 and 2023, 86 fetuses with one or more anomalies identified on obstetric ultrasound underwent HTS (primary targeted gene panel and then ES), allowing making a definite molecular diagnosis in 28% of cases (24/86) (23 families affected). The value of such an analysis in antenatal care has already been demonstrated in the literature (Fu et al., 2018; Lord et al., 2019; Petrovski et al., 2019; Normand et al., 2018; Boissel et al., 2018; Tran Mau-Them et al., 2023; Lefebvre et al., 2021; Gabriel et al., 2022; Fu et al., 2022), despite all the difficulties inherent in interpreting antenatal data in practice (insufficient literature data on antenatal phenotypes, incomplete phenotypes, dual diagnosis, interpretations of VUSs and IF, limited time).

The question of whether or not to continue the pregnancy is of paramount importance in prenatal diagnosis. It should be discussed before performing the test. As previously described (Kalynchuk et al., 2015), the majority of the couples considered that the molecular results would not affect their decision to continue the pregnancy, with 77% of pregnancies terminated before the molecular results were available.

In addition to its benefits for family management, this work will allow better describing and categorizing certain antenatal phenotypes, with the aim of enriching antenatal data on both rare and well-known syndromes (e.g., *DDX3X*, *CREBBP*, etc.). A re-analysis of these ES data, based in particular on the analysis of new phenotypes, postnatal follow-up data or the classification of VUSs over time, would seem relevant (Fu et al., 2022; Mone et al., 2022), even if a postnatal exome or genome sequencing would certainly allow more molecular diagnoses to be made (Thauvin-Robinet et al., 2025). In the future, it would be interesting to gather these antenatal clinical and molecular data, in order to create a searchable clinical database of antenatal gene expression and to enrich the antenatal OMIM phenotypes with clinical entities that are well known postnatally.

## Data Availability

The data that support the findings of this study are available on request from the corresponding author. The data are not publicly available due to privacy and ethical restrictions.
