# Supplementary material for: Evaluation of the contribution of trio-exome sequencing in selected prenatal indications
Source: Front Genet. 2026 May 11;17:1761449. doi: 10.3389/fgene.2026.1761449 (PMC13198924; doi:10.3389/fgene.2026.1761449)
Supplement: Supplementary file 2 [file Table1.docx]

**Supplementary Table 1: Genes selected for each targeted gene panel.**

| List of Vermian hypoplasia genes   \| **AHI1** \| **CSPP1** \| **NPHP1** \| **SNX14** \| \| --- \| --- \| --- \| --- \| \| **AMPD2** \| **EMC1** \| **OFD1** \| **SPTBN2** \| \| **ARL13B** \| **ERCC6** \| **OPHN1** \| **STXBP1** \| \| **ARX** \| **EXOSC3** \| **PCLO** \| **TCTN1** \| \| **ATP2B3** \| **EXOSC8** \| **PDYN** \| **TCTN2** \| \| **B9D1** \| **FKRP** \| **PIGS** \| **TCTN3** \| \| **B9D2** \| **FKTN** \| **PIGT** \| **THOC2** \| \| **BRAT1** \| **FOXC1** \| **PLA2G6** \| **TMEM107** \| \| **BRF1** \| **GOSR2** \| **PMM2** \| **TMEM138** \| \| **C2CD3** \| **GPR56** \| **PMOMT** \| **TMEM17** \| \| **C5ORF42** \| **GPSM2** \| **POMGNT1** \| **TMEM216** \| \| **CACNA1A** \| **GRID2** \| **POMT1** \| **TMEM231** \| \| **CACNA1G** \| **GTDC2** \| **POMT2** \| **TMEM237** \| \| **CACNA2D2** \| **IFT172** \| **PRKCG** \| **TMEM240** \| \| **CASK** \| **INPP4A** \| **PRUNE1** \| **TMEM67** \| \| **CC2D2A** \| **INPP5E** \| **PTF1A** \| **TSEN2** \| \| **CCDC22** \| **ISPD** \| **RARS2** \| **TSEN34** \| \| **CEP104** \| **ITPR1** \| **RELN** \| **TSEN54** \| \| **CEP120** \| **KCNC3** \| **ROBO3** \| **TUBA1A** \| \| **CEP290** \| **KIAA0556** \| **RPGRIP1L** \| **VLDLR** \| \| **CEP41** \| **KIAA0586** \| **SACS** \| **VRK1** \| \| **CHD7** \| **KIF1A** \| **SIL1** \| **WDR73** \| \| **CHMP1A** \| **KIF7** \| **SKOR2** \| **WDR81** \| \| **CLP1** \| **LAMA1** \| **SLC2A1** \| **WNT1** \| \| **COQ8A** \| **LARGE** \| **SLC9A1** \| **ZIC1** \| \| **CPLANE1** \| **MKS1** \| **SLC9A6** \| **ZIC4** \| | List of Abnormal gyration genes   \| **ACTB** \| **EML1** \| **NEDD4L** \| **SNAP29** \| \| --- \| --- \| --- \| --- \| \| **ACTG1** \| **EOMES** \| **NPRL3** \| **TBC1D23** \| \| **ADSL** \| **ERMARD** \| **NSDHL** \| **TBR2** \| \| **AKT3** \| **EXOSC3** \| **OCLN** \| **TOE1** \| \| **AMPD2** \| **EXOSC8** \| **PAFAH1B1(LIS1)** \| **TSEN15** \| \| **ARFGEF2** \| **EZH2** \| **PAX6** \| **TSEN2** \| \| **ARX** \| **FAT4** \| **PCLO** \| **TSEN34** \| \| **ASNS** \| **FH** \| **PDHA1** \| **TSEN54** \| \| **B3GALNT2** \| **FIG4** \| **PIK3CA** \| **TUBA1A** \| \| **B4GAT1** \| **FKRP** \| **PIK3R2** \| **TUBA8** \| \| **CASK** \| **FKTN** \| **POMGNT1** \| **TUBB(TUBB5)** \| \| **CCND2** \| **FLNA** \| **POMGNT2** \| **TUBB2B** \| \| **CDK5** \| **FOXG1** \| **POMK** \| **TUBB3** \| \| **CDKL5** \| **GMPPB** \| **POMT1** \| **TUBG1** \| \| **CHD7** \| **GPR56** \| **POMT2** \| **VLDR** \| \| **CHMP1A** \| **GRIN1** \| **PTEN** \| **VPS53** \| \| **CLP1** \| **K1AA1279** \| **RAB18** \| **VRK1** \| \| **COL18A1** \| **KIAA1109** \| **RAB3GAP1** \| **WDR62** \| \| **CRPPA (ISPD)** \| **KIF2A** \| **RAB3GAP2** \| **WDR81** \| \| **CUL4B** \| **KIF5C** \| **RARS2** \|  \| \| **DAG1** \| **LARGE1** \| **RELN** \|  \| \| **DCHS1** \| **LRP2** \| **RTTN** \|  \| \| **DCX** \| **MAST1** \| **RXYLT1 (TMEM5)** \|  \| \| **DEPDC5** \| **MTOR** \| **SEPSECS** \|  \| \| **DYNC1H1** \| **NDE1** \| **SLC25A46** \| \| |
| --- | --- | --- | --- | --- | --- | --- | --- | --- | --- | --- | --- | --- | --- | --- | --- | --- | --- | --- | --- | --- | --- | --- | --- | --- | --- | --- | --- | --- | --- | --- | --- | --- | --- | --- | --- | --- | --- | --- | --- | --- | --- | --- | --- | --- | --- | --- | --- | --- | --- | --- | --- | --- | --- | --- | --- | --- | --- | --- | --- | --- | --- | --- | --- | --- | --- | --- | --- | --- | --- | --- | --- | --- | --- | --- | --- | --- | --- | --- | --- | --- | --- | --- | --- | --- | --- | --- | --- | --- | --- | --- | --- | --- | --- | --- | --- | --- | --- | --- | --- | --- | --- | --- | --- | --- | --- | --- | --- | --- | --- | --- | --- | --- | --- | --- | --- | --- | --- | --- | --- | --- | --- | --- | --- | --- | --- | --- | --- | --- | --- | --- | --- | --- | --- | --- | --- | --- | --- | --- | --- | --- | --- | --- | --- | --- | --- | --- | --- | --- | --- | --- | --- | --- | --- | --- | --- | --- | --- | --- | --- | --- | --- | --- | --- | --- | --- | --- | --- | --- | --- | --- | --- | --- | --- | --- | --- | --- | --- | --- | --- | --- | --- | --- | --- | --- | --- | --- | --- | --- | --- | --- | --- | --- | --- | --- | --- | --- | --- | --- | --- | --- | --- | --- | --- | --- | --- |
| List of large hyperechogenic kidneys without urinary tract abnormalities genes   \| **AHI1** \| **CEP41** \| **KIAA0586** \| **TMEM138** \| \| --- \| --- \| --- \| --- \| \| **ALMS1** \| **CEP83** \| **KIF14** \| **TMEM216** \| \| **ANKS3** \| **CSPP1** \| **LZTFL1** \| **TMEM231** \| \| **ANKS6** \| **DCDC2** \| **MAPKBP1** \| **TMEM237** \| \| **ARL13B** \| **DDX59** \| **MKKS** \| **TMEM67** \| \| **ARL6** \| **DNAJB11** \| **MKS1** \| **TRAF3IP1** \| \| **B9D1** \| **DYNC2H1** \| **NEK1** \| **TRIM32** \| \| **B9D2** \| **DZIP1L** \| **NEK8** \| **TTC21B** \| \| **BBIP1** \| **EVC** \| **NPHP1** \| **TTC8** \| \| **BBS1** \| **EVC2** \| **NPHP3** \| **WDPCP** \| \| **BBS10** \| **EXOC4** \| **NPHP4** \| **WDR34** \| \| **BBS12** \| **FAN1** \| **OFD1** \| **WDR35** \| \| **BBS2** \| **GANAB** \| **PDE6D** \| **WDR60** \| \| **BBS4** \| **GLIS2** \| **PKD1** \| **XPNPEP3** \| \| **BBS5** \| **HNF1B** \| **PKD2** \| **ZNF423** \| \| **BBS7** \| **IFT122** \| **PKHD1** \|  \| \| **BBS9** \| **IFT140** \| **RPGRIP1L** \|  \| \| **C8orf37** \| **IFT172** \| **SCLT1** \|  \| \| **C2CD3** \| **IFT27** \| **SDCCAG8** \|  \| \| **PCARE** \| **IFT43** \| **SLC41A1** \|  \| \| **CPLANE1** \| **IFT80** \| **TBC1D32** \|  \| \| **CC2D2A** \| **IFT81** \| **TCTN1** \|  \| \| **CEP164** \| **INPP5E** \| **TCTN2** \|  \| \| **CEP19** \| **INVS** \| **TCTN3** \|  \| \| **CEP290** \| **IQCB1** \| **TMEM107** \|  \| | List of ophthalmologic issues genes (microphthalmia and vitreous hyperplasia)   \| **ABCB6** \| **COL4A1** \| **FRS2** \| **MSX2** \| **PRSS56** \| **SOX14** \| \| --- \| --- \| --- \| --- \| --- \| --- \| \| **ACTB** \| **COX7B** \| **FZD4** \| **MYOC** \| **PTCH1** \| **SOX2** \| \| **ACTG1** \| **CPAMD8** \| **FZD5** \| **MYRF** \| **PXDN** \| **SOX21** \| \| **ADAMTS10** \| **CREBBP** \| **GALT** \| **NAA10** \| **RAB18** \| **SRD5A3** \| \| **ADAMTS17** \| **CRYAA** \| **GDF3** \| **NDP** \| **RAB3GAP1** \| **STRA6** \| \| **ADAMTS18** \| **CRYAB** \| **GDF6** \| **NDUFB11** \| **RAB3GAP2** \| **TBC1D20** \| \| **AHR** \| **CRYBA1** \| **GJA1** \| **NHS** \| **RARB** \| **TEK** \| \| **ALDH1A3** \| **CRYBB1** \| **GJA8** \| **NOTCH1** \| **RAX** \| **TENM3** \| \| **ATOH7** \| **CRYBB2** \| **HCCS** \| **NUP188** \| **RBP4** \| **TFAP2A** \| \| **B3GALTL** \| **CRYBB3** \| **HESX1** \| **OCRL** \| **SALL1** \| **TMEM98** \| \| **BCOR** \| **CRYGD** \| **HMGB3** \| **OTX2** \| **SALL2** \| **TMEM98** \| \| **BEST1** \| **CYP1B1** \| **HMX1** \| **PACS1** \| **SEMA3E** \| **TMX3** \| \| **BFSP1** \| **CYP27A1** \| **HSF4** \| **PACS1** \| **SHH** \| **TRIM44** \| \| **BFSP2** \| **EDNRB** \| **IGBP1** \| **PAX2** \| **SIL1** \| **VAX1** \| \| **BHLHE40** \| **EP300** \| **IPO13** \| **PAX3** \| **SIMO** \| **VAX2** \| \| **BMP4** \| **EPHA2** \| **ITPR1** \| **PAX6** \| **SIX3** \| **VCAN** \| \| **BMP7** \| **EYA1** \| **LARGE1** \| **PDE6D** \| **SIX6** \| **VIM** \| \| **C12orf57** \| **FBXW11** \| **LIM2** \| **PIGL** \| **SLC16A12** \| **VRK2** \| \| **CAPN15** \| **FNBP4** \| **LRP5** \| **PITX2** \| **SLC25A24** \| **VSX2** \| \| **CDH2** \| **FOXC1** \| **LTBP2** \| **PITX3** \| **SLC38A8** \| **WDR37** \| \| **CENPF** \| **FOXC2** \| **MAB21L2** \| **POMGNT1** \| **SLC38A8** \| **WNT7b** \| \| **CHD7** \| **FOXE3** \| **MAF** \| **POMK** \| **SMCHD1** \| **WT1** \| \| **CHMP4B** \| **FRAS1** \| **MFRP** \| **POMT2** \| **SMO** \| **YAP1** \| \| **CHRDL1** \| **FREM1** \| **MIP** \| **PORCN** \| **SMOC1** \| **ZEB2** \| \| **CNBP** \| **FRMD7** \| **MITF** \| **PQBP1** \| **SOX10** \| **ZIC5** \| |
| List of midline defect genes excluding agenesis of the corpus callosum   \| **DISP1** \| **SIX3** \| \| --- \| --- \| \| **DLL1** \| **SUFU** \| \| **FGF8** \| **TGIF1** \| \| **FGFR1** \| **ZIC2** \| \| **FOXH1** \|  \| \| **GLI2** \|  \| \| **NODAL** \|  \| \| **SHH** \|  \| | List of agenesis of the corpus callosum genes  List of 2,788 genes known to be involved in intellectual disability grouped in the SysNDD database.  SysNDD list: https://www.sysndd.dbmr.unibe.ch/table/overview  List of polymalformative syndrome and hygroma colli-generalized hydrops genes:  This list contains all the genes referenced on OMIM as involved in human disorders (OMIM morbid genes), i.e. 4,692 genes.  OMIMome list: <https://www.omim.org/> |
