# Supplementary material for: Evaluation of the contribution of trio-exome sequencing in selected prenatal indications
Source: Front Genet. 2026 May 11;17:1761449. doi: 10.3389/fgene.2026.1761449 (PMC13198924; doi:10.3389/fgene.2026.1761449)
Supplement: Supplementary file 3 [file DataSheet1.docx]

SUPPLEMENTAL DATAS

**Supplemental data 1: Diagnostic yield of exome sequencing in 1287 fetuses (1-5).**

For this non-exhaustive review of the literature, five studies (Lord et al. 2019, Petrovski et al. 2019, Fu et al. 2018, Normand et al. 2018, Boissel et al. 2018) were selected due to the large size of their respective cohorts, the diversity of inclusion criteria, and the accuracy of clinical and molecular informations. The overall diagnostic yield was calculated by averaging the overall diagnostic yield of each study, even though it is very difficult to compare cohorts due to multiple criteria influencing the results.

|  | **Lord et al. 2019** | **Petrovski et al. 2019** | **Fu et al. 2018** | **Normand et al. 2018** | **Boissel et al. 2018** | **Total** |
| --- | --- | --- | --- | --- | --- | --- |
| **Number of fetuses** | **610** | **234** | **196** | **146** | **101** | **1287** |
| **Total diagnostic yield in the cohort** | **8,50%** | **10%** | **24%** | **32%** | **19%** | **19%** |
| **Diagnostic yield by indication** | | | | | | |
| **Muscular and/or musculoskeletal anomaly** | NA | NA | NA | 39%  (28/72) | 25%  (2/8) | 32% |
| **Urogenital/genitourinary anomaly** | NA | NA | 23.1% (6/26) | 32%  (12/38) | NA | 27.5% |
| **Renal agenesis** | NA | NA | NA | NA | 80%  (2/8) | 25% |
| **Lymphatic anomaly or effusion** | NA | 24%  (5/21) | NA | NA | NA | 24% |
| **Nuchal translucency** | 3.2%  (3/93) | 12%  (6/51) | NA | NA | NA | 23.6% |
| **Skeletal anomaly** | 15.4% (10/65) | 24%  (8/34) | 30%  (3/10) | NA | NA | 23.1% |
| **Others (see article)** | NA | 10%  (3/29) | NA | 36%  (27/75) | NA | 23% |
| **(Central) Nervous system anomaly** | 3%  (2/69) | 22% (11/49) | 23.1% (15/65) | 34%  (22/65) | 19%  (7/36) | 20.2% |
| **Craniofacial anomaly** | 3.1%  (1/32) | 0%  (0/3) | 23.5% (4/17) | 46%  (22/48) | NA | 18.5% |
| **Cardiac/cardiovascular anomaly** | 11.1% (9/81) | 5%  (4/77) | 20.6% (7/34) | 30%  (11/37) | NA | 16.6% |
| **Pulmonary and/or diaphragm anomaly** | 0%  (0/23) | 0%  (0/2) | NA | 43%  (9/21) | NA | 14.3% |
| **VACTERL** | NA | NA | NA | NA | 11%  (1/9) | 11% |
| **Hydrops** | 9%  (3/33) | NA | NA | NA | NA | 9% |
| **Renal anomaly** | 0%  (0/16) | 16%  (4/25) | NA | NA | 0%  (0/11) | 5.3% |
| **Spinal anomaly** | 10%  (1/10) | 0%  (0/1) | NA | NA | NA | 5% |
| **Gastrointestinal anomaly** | 2%  (1/45) | 0%  (0/6) | 0%  (0/5) | NA | NA | 0.6% |

*NA : not available.*

There is heterogeneity in the different diagnostic yields for the same signs between different cohorts. This can be explained by the fact that the cohorts are small and by the diversity of inclusion criteria and the grouping of clinical signs, which sometimes differ from one study to another. For example, some studies grouped together muscular and skeletal malformations, while others considered only skeletal anomalies.

**REFERENCES**

1. Lord J, McMullan DJ, Eberhardt RY, Rinck G, Hamilton SJ, Quinlan-Jones E, et al. Prenatal exome sequencing analysis in fetal structural anomalies detected by ultrasonography (PAGE): a cohort study. The Lancet. 2019 Feb;393(10173):747–57.

2. Petrovski S, Aggarwal V, Giordano JL, Stosic M, Wou K, Bier L, et al. Whole-exome sequencing in the evaluation of fetal structural anomalies: a prospective cohort study. The Lancet. 2019 Feb;393(10173):758–67.

3. Fu F, Li R, Li Y, Nie Z ‐Q., Lei T, Wang D, et al. Whole exome sequencing as a diagnostic adjunct to clinical testing in fetuses with structural abnormalities. Ultrasound in Obstet & Gyne. 2018 Apr;51(4):493–502.

4. Normand EA, Braxton A, Nassef S, Ward PA, Vetrini F, He W, et al. Clinical exome sequencing for fetuses with ultrasound abnormalities and a suspected Mendelian disorder. Genome Med [Internet]. 2018 Dec [cited 2024 Sep 17];10(1). Available from: https://genomemedicine.biomedcentral.com/articles/10.1186/s13073-018-0582-x

5. Boissel S, Fallet-Bianco C, Chitayat D, Kremer V, Nassif C, Rypens F, et al. Genomic study of severe fetal anomalies and discovery of GREB1L mutations in renal agenesis. Genetics in Medicine. 2018 Jul;20(7):745–53.

**Supplemental data 2: Materials and methods**

**Selected indications: details regarding brain anomalies**

Prenatal images of vermian hypoplasia cases were reviewed by the expert center in Trousseau Hospital (APHP, Paris). Prenatal images of abnormal gyration cases with moderate or focal involvement were reviewed by an expert practitioner in Strasbourg. Prenatal images of cases with midline defects were reviewed by the expert center of Rennes University Hospital.

**Exome sequencing (ES)**

ES was performed and analyzed in the Genetic Diagnostics laboratory of Strasbourg University Hospital. The series of samples were processed for trio sequencing, with each flow cell containing 9 samples corresponding to three families (samples from the fetus, mother and father), except for 50 families where parental samples were pooled, allowing some flow cells to contain samples corresponding to five families (sample from the fetus and pooled sample from the parents). The amount of DNA in pooled parental samples was two times higher than that in unpooled parental samples to improve the detection of inherited variations.

***Library preparation***

- Enzymatic fragmentation was performed using Twist Library Preparation EF Kit 1 or SureSelect Enzymatic Fragmentation Kit for ILM. The DNA was diluted in this enzyme mixture and placed in a thermocycler for 22 minutes at 32°C for Twist kit, and 10 minutes at 37°C for SureSelect kit, yielding DNA fragments of 300-400 bp in length. The enzymes were then inactivated by increasing the temperature to 65°C.
- End-Repair and A-Tailing: a few nucleotides were added to clean up the cohesive ends of the fragments obtained, as well as ‘poly-A tails’.
- Universal adapters were ligated to the ends of the poly-A tails.
- Pre-capture amplification: primers complementary to the adapters and containing the index sequences for patients’ identification and P5 and P7 ends that allowed their binding to the flow cell were used.
- The DNA fragments of interest were purified using magnetic beads.
- Pre-capture libraries were verified using a bioanalyzer: a uniform peak of 375-425 bp should be present for Twist libraries, and 330-450 bp for SureSelect libraries, indicating the correct length of the fragments obtained. The amount of DNA required was 80 ng/µL for the Twist kit and 90 ng for the SureSelect kit.
- DNA library pool: Trio-ES was performed (fetus and both parents). For 50 fetuses, the maternal and paternal DNAs were pooled allowing loading 5 index cases instead 3 on the same flow cell. DNAs were pooled in equimolar quantities.
- Biotinylated DNA probes complementary to the regions of interest to be sequenced (exons coding for the exome) were hybridized with fragmented DNA samples.
- Biotinylated DNA libraries were captured using streptavidin-coated beads.
- Post-capture amplification: each DNA fragment was cloned to amplify the signal during sequencing.

***Clusterization***

DNA fragments were hybridized to the flow cell via adapters ligated to their ends. Several cycles of bridge PCR were needed to amplify DNA fragments and create clusters on the flow cell for sequencing.

***Massive parallel sequencing***

The complementary strand of each DNA fragment hybridized on the flow cell was synthesized. Each time a nucleotide was incorporated, a fluorescent signal was emitted and detected. At the end of sequencing, all DNA fragments were sequenced in parallel, yielding the sequences of interest of the whole exome, in the form of fluorescent signals. All data were saved in .bcl file format.

**Bioinformatics analysis**

After sequencing, the reads generated (800 million reads) were analyzed using the STARK (Stellar Tools from raw sequencing data Analysis to variant RanKing) pipeline developed in Strasbourg University Hospital. This pipeline was used to process the raw data from ES to identify likely pathogenic variants. The various stages are summarized below:

- Demultiplexing of reads to associate each read with a specific patient sample.
- Generation of a sequence quality score (Phred) and filtering of sequences with a quality below a set threshold,
- Alignment of reads to the whole genome: positioning of the DNA sequence reads generated against the reference sequence using the Burrows-Wheeler Aligner (BWA) tool^12^. A .bam file was generated.
- Identification of sequence variations between the generated sequence and the reference sequence, using the Genome Analysis Toolkit (GATK)^12^ for point variants.
- Variant calling for point variants (SNVs, indels) using HaplotypeCaller and then GenotypeGVCFs. This information was compiled in a .vcf file containing variants classified as ‘true positive’.
- Annotation of point variants using Varank^13^: all available information on each of the variants identified was gathered and annotated with information allowing their ranking according to their pathogenic risk (score calculation, comparison with databases, etc.). Using this information, biologists were able to analyze their pathogenicity by applying filters to sort and prioritize them.
- CANOES^14^ was used to detect CNVs and normalize sequencing depths across all samples within the run from the .bam files, and then to identify deviations in depth at given positions, and detect any gains or losses of copies of genomic segments. The changes detected were then annotated using the AnnotSv^15^ algorithm.

For the samples from fetuses with polymalformative syndromes, persistent hygroma colli and hydrops, the analysis was performed using the OMIMome targeted gene panel (set of morbid genes from the OMIM database). For the samples from fetuses with ACC, the Sys NDD list was used.

***Identitovigilance***

All analyzes of sequencing data began with identity verification. To do so, some SNPs (rs3735803, rs2306331, rs7301328, rs1047179) were genotyped by PCR using Taqman probes in parallel with their ES data analysis. The SNPs studied were selected based on their location - four on autosomes and one on gonosomes (*SRY*) - and their minor allele frequency close to 0.5 in the French population. Once the data from the two methods were available, the different alleles were compared at the different loci selected. This analysis reduced the risk of two different individuals having the same profile at all these loci to 3% (0.5^5). The concordance of the fetus/parent trio was assessed by examining the number of *de novo* variations at the end of the trio analysis.
